# Mr. Giraud's Cass of Monstrosity

**Published:** 1803-08-01

**Authors:** J. T. Giraud

**Affiliations:** Faversham


### *To the Editors of the Medical and Physical Journal.*

GENTLEMEN,

THE following singular case of Monstrosity has lately come under my observation; and although the particulars of it, so far as regard the child, may not afford any practical inference, yet as it may be interesting to practitioners of midwifery, if you think it worth a place in your very useful work, it is at your service.

I am, yours, &c.

J. T. GIRAUD.

*Faversham,*

*Nov. 8, 1802.*

Mrs.

Mrs. B. aged 33 years, having borne five healthy children, was taken in labour the beginning of June last, the sixth time. The presentation was natural, and the birth of the child easy, till it arrived at the hips, when, in addition to the natural pains, a very considerable force was found necessary to complete the delivery. It proved that this extraordinary difficulty was occasioned by a preternatuaal tumour, in bulk, at least, equal to the child's head, somewhat of a pyramidal form, the base attached to, and extending over the glutæi muscles. The child was a full grown female fœtus, strong and healthy, and in every other respect well formed. The tumour had an unequal feel, some parts possessing a considerable degree of firmness, while other parts were soft, with apparent fluctuation. A few days after birth, a portion of the surface inflamed and ulcerated, discharging a considerable quantity of thin sanious matter, the size and shape of the tumour undergoing, in consequence, some degree of alteration. This ulceration and discharge continued, more or less, upwards of three months, without in the least impairing the health of the child, or seeming to cause any great inconvenience. At length, however, the ulceration became more deep and extensive, the health of the child became impaired, and it gradually sunk. At the time of death, a considerable alteration had taken place, both in the form and proportionable size of the tumour, and sphacelation had commenced, and extended over a considerable portion of the surface. The annexed engraving, from a sketch taken after death, will give a pretty correct idea of the form and proportion of the tumour at that time.

Upon dissection, the tumour was found to be constituted of innumerable cysts, of various sizes, formed of thickened cellular membrane, and intermixed with adipose matter; their contents were various, and put on the different forms of lymph, glairy fluid, pus, and grumous matter, of a deep yellow and brown colour. I sent a part of this swelling to Mr. A. Cooper, who saw the child, and was consulted respecting the propriety of an operation for the removal of the tumour. During gestation the mother hadsuffered no unusual inconvenience, neither had she experienced any fright, nor laboured under any disagreeable impression. Her family, however, had some striking traits, the most conspicuous of which indicated a scrofulous diathesis, and to which, perhaps, the morbid state of the above described subject, may be referred. The following is a brief statement of these facts.

G. W.

**G. W.**

At an early age lost a leg, in consequence of a diseased knee joint; he enjoyed good health, however, to an advanced age.

**His Wife, Mrs. W.**

Had one thigh considerably shorter than the other. She used to assert that her father and grandfather laboured under the same defect. She bore many children, and lived to upwards of ninety years.

**Of their Children,**

**W.**

Now living, has been many years subject to nephritic and calculous complaints, and rheumatism: His —

**J.**

Now living, has been afflicted with scrofulous tumours and ulcerations.

**G.**

Died, aged about sixty, of schirrous intestinum rectum.

**P.**

Lost his sight at the age of 15 months, from inflammation induced by measles, shortly succeeded by scarlet fever.

**G.**

Died in the prime of life, after long lingering under lumbar abscess.

**Son,**

Has several children, some of whom are subjects of scrofula, and one of his daughters has a short thigh.

**Daughter,**

Who is the mother of the subject above described, has the same shortness of one thigh, which her grandmother and others of her ancestors laboured under.

